# Variability in laboratory parameters used for management of Cushing’s syndrome

**DOI:** 10.1007/s12020-015-0676-9

**Published:** 2015-07-10

**Authors:** Francesca Pecori Giraldi, Alberto G. Ambrogio

**Affiliations:** Department of Clinical Sciences and Community Health, University of Milan, Milan, Italy; Neuroendocrinology Research Laboratory, Istituto Auxologico Italiano, Via Zucchi 18, 20095 Milan, MI Italy

**Keywords:** Cortisol, ACTH, Diagnosis, Cushing’s syndrome, Cushing’s disease, Assay

## Abstract

The progress in assay methodology, from the use of radioactive tracers to chemiluminescent signals, from competitive to chromatographic techniques and from serum or urine to saliva has considerably impacted on hormonal measurements. The clinician now may choose among multiple tests but the inherent variability in cortisol and ACTH secretion, coupled to lack of harmonization among assay procedures and normal ranges mandates careful interpretation of any result. The present review will examine factors which affect interpretation of cortisol and ACTH measurements and their impact on tests used for management of Cushing’s syndrome.

The management of Cushing’s syndrome rests on the determination of the two main hormones of the hypothalamic-pituitary-adrenal (HPA) axis, namely cortisol and ACTH. These may be measured in serum and urine, in the early morning, at night, or over 24 h as well as at baseline or after specific challenges. The endocrinologist is called to interpret the resulting hormone measurements on the basis of a large body of studies describing cut-offs to be used in order to exclude or confirm the initial clinical suspicion and establish the etiology of hypercortisolism. In all these situations, clinicians rely heavily on assay results, i.e., a number which represents hormone concentration. This number, however, has to be interpreted taking into account the fact that both cortisol and ACTH are secreted with considerable circadian and ultradian variation and, further, commutability of assays performed in large-scale non-endocrine laboratories may not meet requirements for fine endocrine diagnosis, e.g., overestimation, non-linear bias. Indeed, the gap between assay procedures and diagnostic criteria established at referral centers and the results of field assays used in routine clinical practice—which are mostly chosen on economic and logistical basis—is widening. In this context, it is worth recalling that although Cushing’s syndrome is a rare disorder and usually managed by dedicated endocrinologists, the burden of the initial suspicion and diagnostic work-up rests squarely on the general endocrine clinic thus both practitioners will be called into play.

Several factors are likely to contribute to the variability in cortisol and ACTH measurements. The present paper will discuss these issues and their impact on tests used for the management of Cushing’s syndrome in order to provide an up-to-date review for the general and specialist endocrinologist alike.

## Urinary free cortisol

Determination of urinary free cortisol (UFC) is historically the premier measure for the diagnosis of Cushing’s syndrome but its reliability has come to be questioned in the past few years [1]. Further, although severity of Cushing’s syndrome is often gauged by UFC levels and patients with extremely high levels are prone to develop the most severe complications [2, 3], a strict correlation between UFC concentrations and clinical signs of hypercortisolism may not be readily detectable [4]. This impacts the interpretation of treatment responses and detection of disease recurrence, as changes in UFC are not always accompanied by parallel amelioration or worsening of clinical features.

Several factors are responsible for the increasing doubts as to the reliability of UFC as a marker of endogenous hypercortisolism. One of the key problems is likely to be assay-related as methods for measuring UFC have changed considerably over the past decades but problems related to cortisol metabolites and conjugates continue to interfere with accurate measurement of cortisol itself [5]. In immunoassays, with either radioactive or chemiluminescent tracers, antibodies are raised against protein-conjugated cortisol and antibody specificity inevitably varies [6]. Many cortisol metabolites, e.g., free or conjugated tetrahydro-, 20dihydro-, 6ß-hydrocortisol/cortisone, and cortols, as well as other as yet unidentified steroids, are secreted in urine and may cross-react with the antibody [7]. Cross-reactivity with 11-deoxycortisol must be excluded in patients on metyrapone, as levels of this steroid increase due to 11ß-hydroxylase blockade [8]. Metabolites are usually excreted in far greater amounts than cortisol itself, thus even small interferences will translate into gross overestimation of “urinary free cortisol.” Solvent extraction with dichloromethane removes most polar cortisol metabolites, e.g., glucuronides and sulfates, thereby reducing values by two or three times [9]; indeed, upper limits of the normal range reported by direct urine assays are roughly twice as high as those reported in extracted urine (approx. 150 µg/24 h or 410 nmol/l24 h vs 80 µg/24 h or 220 nmol/24 h, respectively). Chromatographic methods, i.e., high-performance liquid chromatography, liquid, or gas chromatography followed by mass or tandem mass spectrometry, are currently advocated as the most accurate means of measuring cortisol in urine [10] but are as yet not widely available both in terms of equipment and technical expertise. This obviously represents a drawback for routine clinical practice. Chromatographic methods achieve greater specificity, in fact cortisol measurements are roughly half those reported by immunoassays [11], but are subject to interferences (“matrix effects”) which may affect accuracy and reproducibility [12]. Altogether, quality assessment programs revealed from 20 to nearly 60 % interassay variability in routine UFC measurements [13].

**Fig. 1:**
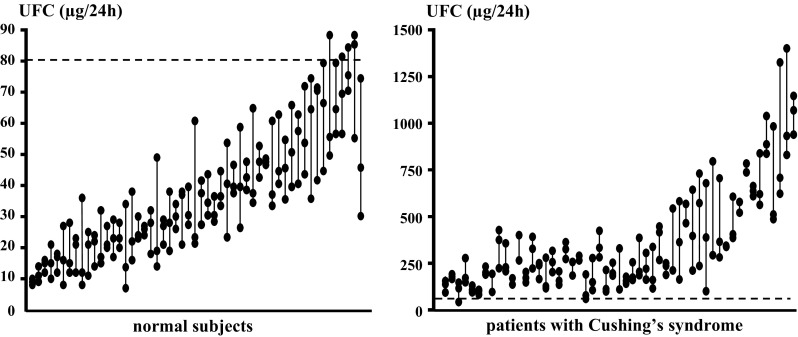
Variability of urinary free cortisol measurements (UFC). Results of measurements on three successive 24 h collections in healthy individuals (*left panel*) and patients with Cushing’s syndrome (*right panel*). Each set of three connected dots represents an individual. Dashed line is set at 80 µg/24 h, i.e., the upper limit of the normal range for post-extraction radioimmunoassay (Coat-a-Count, Diagnostic Products Corp, Los Angeles, U.S.A)

It should also be recalled that cortisol is secreted in a highly pulsatile fashion, with considerable diurnal fluctuations. Day-to-day variability of UFC has been estimated around 40 % [14] and excursions may be even larger in patients with Cushing’s syndrome [4, 15] (Fig. 1). This obviously does not allow clinicians to rely on a single determination as a comprehensive measure of hypercortisolism or even the mean of three [4]. In fact, it has been estimated that up to ten measurements are required to achieve reliability of the mean value [16]. This has to be taken into account when repeat UFC measurements are performed, for example, to interpret response to medical treatments [17, 18] or to follow progression of recurrence [19] (Table 1). Obviously, cyclical Cushing’s syndrome represents an additional cause of variable assay results.

**Table 1:** Issues associated with hormonal assays used in the management of Cushing’s syndrome

Parameter	Sampling	Use	Specific issues	Possible solutions
Urinary free cortisol	Circadian secretion After 8 mg dexamethasone	Diagnosis Response to treatment Follow-up	Interference due to cortisol metabolites	Urine extraction, chromatographic assays
			Completeness of 24 h urine collection	Urinary creatinine
			Interferences due to medications	Case history
			Day-to-day variability	Multiple sampling
			Gender	Sex-specific normal ranges
Salivary cortisol	Late evening	Diagnosis Response to treatment Follow-up	Assay-related variability Aging Day-to-day variability	Assay standardization Age-adjusted ranges Multiple sampling
Serum cortisol	Morning	After surgery	Increased CBG, e.g., contraceptives	Case history
			Biological variability	Multiple sampling
			Gender	Sex-specific ranges
	Late evening	Diagnosis	Increased CBG Aging	
	After low and high dose dexamethasone	Diagnosis Response to treatment Follow-up	Dexamethasone bioavailability, clearance	Plasma dexamethasone assay
			GR polymorphisms	*GR* gene analysis
			Increased CBG, e.g., contraceptives	Case history
			Aging	Age-adjusted ranges
			Assay variability	Assay-specific cut-offs
	After CRH stimulation	Differential diagnosis	Increased CBG	Case history
Plasma ACTH	Morning	Differential diagnosis	Pulsatility, short half-life Assay-related variability	Multiple sampling Assay standardization
	After CRH stimulation During IPSS	Differential diagnosis	No specific issue	

*CRH* corticotropin-releasing hormone, *IPSS* inferior petrosal sinus sampling, *CBG* cortisol-binding globulin, *GR* glucocorticoid receptor

Male sex is associated with slightly higher UFC levels both in healthy subjects [20] and patients with Cushing’s disease [3]. Increased urinary cortisol metabolites excretion, e.g., tetrahydrocortisol, cortolones, has also been reported in men [21] whereas decreased 11ß-hydroxysteroid dehydrogenase activity is responsible for prevalence of urinary 11-oxo over 11-hydroxymetabolites in women [22]. This difference is maintained with aging, although subtle decreases in UFC can be observed with age [23]. Lastly, as urinary excretion is reliant on renal function, variations in diuresis may affect UFC measurements but this does not appear to occur within physiological changes in urine volume [24]. As compliance in providing full 24-h urine collections must be assured, urinary creatinine is measured to ascertain adequacy of collection; correction for urinary creatinine is usually not necessary for 24 h collections although some laboratories express UFC as ng/mg creatinine.

## Morning serum cortisol

Measurement of morning serum cortisol plays a minor role in the diagnostic work-up of Cushing’s syndrome, as up to 50 % of patients will present values comprised in the normal range [25]. However, several studies have reported a significant correlation between morning serum cortisol levels and specific features in patients with Cushing’s syndrome, e.g., left ventricular mass index [26], hypokalemia, and muscle atrophy [3]. Thus, although morning serum cortisol does not play a significant role in establishing hypercortisolism, it may well represent a parameter for end organ damage.

Conversely, after surgery, morning serum cortisol represents a good predictor for remission and risk of relapse (Table 1), thus accuracy and precision in the low-normal range are necessary. Quality assessment programs report overall good interlaboratory and interkit precision (i.e., <10 %) [27] and interference with other steroids is a lesser problem compared to urinary cortisol. Spuriously elevated cortisol measurements may be recorded in patients on metyrapone due to increased 11ß-deoxycortisol [28] and to increased cortisol-binding globulin (CBG) in patients on mitotane [29].

As regards intraindividual variability, measurements over 20 min in the same subject show good repeatability and it has been estimated that 3 measurements are sufficient to achieve a reliable estimate [16]. However, given the biological variability of morning cortisol around 20 % [30], measurements may differ by ±8 µg/dl (220 nmol/l) over time [16]. Further, seasonal variability impacts women to a greater degree than men with up to 7 µg/dl (200 nmol/l) difference from spring to winter [31]. This is relevant to postsurgical evaluation, as morning serum cortisol is often used to guide weaning off steroid replacement therapy over several months after surgery.

Lastly, morning serum cortisol is higher in healthy men than women [32] and an increase by 20 % has been shown to occur with aging, i.e., from 50- to 80-year old individuals, in both sexes [33].

## Midnight cortisol

In contrast to morning cortisol, late evening or midnight cortisol is clearly altered in patients with Cushing’s syndrome; indeed, absent circadian cortisol rhythmicity is a hallmark of endogenous hypercortisolism. The measurement of cortisol late at night—in serum or saliva—is used as a screening procedure, to follow patients with cyclical hypercortisolism, to establish response to treatment as well as to determine relapse of hypercortisolism after surgery (Table 1).

The standing of midnight cortisol in the diagnostic work-up of Cushing’s syndrome is very much influenced by logistics and health care costs. One the one side, hospitalization in order to sample patients at midnight is not feasible in some countries, on the other side, not all laboratories are equipped to handle salivary specimens, given that its main use, i.e., screening for Cushing’s syndrome, does not justify the expense in high throughput clinical labs. Thus, both approaches are still current.

Age has been shown to affect circadian rhythmicity, as cortisol circadian dipping is blunted with aging [34]. This phenomenon is slowly progressive and begins in the fourth decade of life [23, 35], thus in the age range of Cushing’s syndrome. As low cut-offs are desired to ensure maximal sensitivity of nocturnal cortisol, the number of false positives predictably is higher in 40-year-old and older subjects [23, 35].

Salivary cortisol is subject to some unique concerns [36]. Only unbound cortisol can diffuse into the saliva and, indeed, serum:salivary cortisol ratio is roughly 20:1. One factor which can specifically interfere with salivary cortisol measurement is salivary gland 11ß-hydroxysteroid dehydrogenase type 2 activity which converts cortisol into cortisone and is responsible for 4–6fold higher salivary cortisone vs cortisol concentrations [37]. The abundance of cortisone may prove a significant interference in immunoassays [38], as antibodies raised against cortisol can cross-react with cortisone. In serum, where cortisone is roughly 1/8 with respect to cortisol, cross-reactivity is a negligible problem. Advantages of salivary cortisol are ease of sample collection, e.g., drooling, salivette chewing, long-term storage, and even shipment through regular mail. Drawbacks are the absence of reference preparation, differing reference ranges, and considerable interassay variability. External quality assessment program for salivary cortisol have been set up by several Institutions, e.g., College of American Pathologists, but divergences between assays have already been proven to represent a considerable problem [39]. This translates into the necessity for each lab to develop its own reference range, which is feasible in research setting but not in routine clinical practice; in fact, most clinical laboratories simply adopt technical sheet normal ranges. Lack of harmonization between assays is particularly evident when results obtained from immunoassays are compared with chromatography with the former yielding higher results [40] and the latter susceptible to false positives [41]. Further, conditions such as obesity [40] and diabetes [35] are accompanied by higher midnight salivary cortisol levels per se. Along the same line, salivary cortisol may prove misleading in pseudoCushing [42] or other conditions suspicious for endogenous hypercortisolism [41].

One specific advantage of measuring free cortisol in saliva is that CBG concentrations do not affect salivary steroid ultrafiltration; thus, levels are unaffected by oral contraceptives [37]. Conversely, serum cortisol, i.e., free plus protein-bound cortisol, is inevitably affected by increased CBG levels [43]. While this does not represent a problem for morning serum cortisol values, it certainly affects low, late evening cortisol levels [44]. No significant sex-related difference was observed in serum and salivary midnight cortisol in either patients with Cushing’s syndrome or healthy subjects [45].

**Fig. 2:**
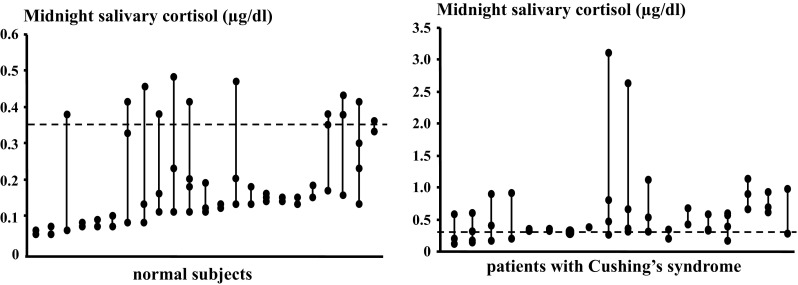
Variability of midnight salivary cortisol measurements. Results of measurements on 2–4 successive collections in healthy individuals (*left panel*) and patients with Cushing’s syndrome (*right panel*). Each set of three connected dots represents an individual. *Dashed line* is set at 0.35 µg/dl, i.e., the upper limit of the normal range (Elecsys, Roche Diagnostics, Mannheim, Germany)

Midnight serum cortisol concentrations over successive nights were proven to be by and large comparable in healthy or hypercortisolemic individuals [46]. Conversely, reproducibility of midnight salivary cortisol over time is not consistent over time; indeed intraindividual variability in measurements was as high as 22 % in healthy individuals, over 30 % in Cushing’s syndrome suspects, and twice as much in patients with Cushing’s syndrome [47, 48] (Fig. 2). Inevitably, this leads to some discordant classification of normal and abnormal values in repeat salivary measurements among subjects suspected of Cushing’s syndrome [41, 48]. This issue becomes particularly relevant in mild Cushing’s syndrome in whom normal salivary values may be recorded repeatedly over time [49] and only a high degree of clinical suspicion leads to the correct diagnosis.

## Dexamethasone suppression tests

Dexamethasone suppression tests play a role in both the diagnosis and the differential diagnosis of Cushing’s syndrome. Low-dose tests (1 mg overnight, i.e., overnight suppression test, OST, or 2 mg over 2 days) are used to identify patients with Cushing’s syndrome whereas high-dose dexamethasone tests (8 or 16 mg) are employed to distinguish between pituitary and ectopic ACTH secretion or to evoke a paradoxical cortisol response in adrenal nodular dysplasia. Low-dose tests are also used to define remission after surgery or medical therapy (Table 1).

For low-dose tests, cortisol is measured in serum, possibly also in saliva, whereas measurements in both serum and 24 h urine are used for the high-dose test. Interpretation of low-dose test requires judging whether the patient’s cortisol concentration is above or below a given diagnostic cut-off; on the other hand, for the high-dose dexamethasone test, pre- and post-dexamethasone cortisol concentrations are compared and the degree of change weighed against specific thresholds. Thus, differences between assays primarily affect the low-dose suppression test, far less than the high-dose suppression test.

The issue of assay-specific cut-offs had been raised already 30 years ago [50] at the time of radioimmunoassays (RIA), but still represents a problem with current chemiluminescent assays [51]. This resulted in higher cortisol levels if patient samples are measured with one or another assay and, thus, the diagnosis “non-suppression” or “suppression” was assay kit-dependent [51]. As regards repeatability of dexamethasone suppression over time, cortisol concentrations with repeat 0.25 mg dexamethasone testing in normal subjects ranged from 50 to 200 % of the first measurement [52]. Discrepancies between test results may prove relevant when OST is performed repeatedly in the same subject, for example in patients with mild hypercortisolism [53], to establish remission after surgery [54] or medical therapy [55] and relapse after successful surgery [56]. Diagnostic accuracy of salivary cortisol after dexamethasone suppression has also been evaluated but specificity appears less than that of serum cortisol [57].

The high-dose dexamethasone test has withstood the test of time less brilliantly than the low-dose test and its suboptimal sensitivity and specificity led some authors advocate its abandonment [1]. However, given the difficulties with CRH supplies and IPSS availability, this test remains the only available means to attempt the differential diagnosis of ACTH-dependent Cushing’s syndrome in several endocrine centers. Assay-dependent differences in dexamethasone-suppressed serum cortisol measurements have been reported for the 8 mg test in normal and pseudoCushing subjects [51]; as the diagnostic criterion for the high-dose test is the percentage decrease from pre-dexamethasone cortisol concentrations, this difference may not be relevant to the diagnostic work-up. As regards urinary cortisol, the same assay-related difficulties reported for baseline UFC apply although intraindividual variability of suppressed cortisol secretion may be less than that of spontaneous concentrations. Reproducibility of cortisol inhibition after high dexamethasone suppression is as yet unknown.

In addition to factors associated with cortisol assays, the response to dexamethasone suppression is affected by bioavailability and clearance of dexamethasone. Dexamethasone plasma concentrations vary considerably in subjects administered the same oral dose [58] and are clearly higher in subjects who suppress than in non-suppressors [59]. This has been shown to occur after both oral and intravenous administration and appears associated with differences in dexamethasone clearance and plasma half-life [59]. The effect of drugs such as phenytoin, rifampicin, and carbamazepine, which accelerate hepatic metabolism, is well known and may lead to false positive results. Measurement of plasma dexamethasone concurrently with serum cortisol has been advocated [60] but proved impractical and too expensive for a screening test in clinical practice.

Aging appears associated with resistance to negative feedback [61] and older subjects are more likely to present unsuppressed cortisol levels; this applies both to normal individuals [50] and Cushing’s syndrome suspects [23], thus increasing the risk for false positives. Weight is inversely correlated with sensitivity to low dose, i.e., 0.05–0.125 mg, dexamethasone inhibition [62] but sensitivity of the 1 mg dexamethasone suppression test appears unaffected by weight [63].

Gender does not appear to play a significant role in sensitivity to dexamethasone suppression [45] although, obviously, women in child-bearing age must not be on oral contraceptives as the increase in CBG may lead to increased false positives [64]. This may particularly be relevant to screening of obese or diabetic patients, as contraceptives are often not perceived to be true medications and may not be reported by patients prior to testing.

Another factor which might affect the sensitivity to dexamethasone suppression are glucocorticoid receptor polymorphisms. Individuals carrying the ER22/23EK variant are more likely to present higher cortisol levels after low-dose dexamethasone suppression whereas carriers of the N363S and Bcl1 polymorphisms present lower cortisol levels [65]. A recent study has shown that the Bcl1 polymorphism does not impact cortisol levels after OST in patients with Cushing’s disease [66] but whether this holds true also for low cortisol levels, i.e., normal individuals or Cushing’s syndrome suspects, remains to be tested.

## Plasma ACTH

Plasma ACTH is pivotal to the differential diagnosis of Cushing’s syndrome, both as regards ACTH-dependency versus ACTH-independency and pituitary versus ectopic ACTH-secreting tumors. ACTH is measured in unchallenged samples or after stimulation with corticotrophin-releasing hormone (CRH) both as a standalone test or during inferior petrosal sinus sampling (IPSS).

**Fig. 3:**
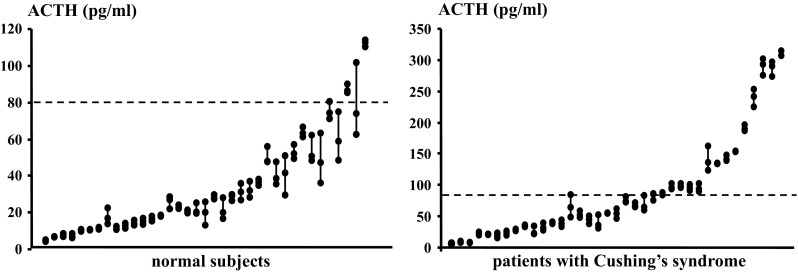
Variability of plasma ACTH measurements. Results of repeat sampling from an indwelling venous catheter over 60 min in healthy individuals (*left panel*) and patients with Cushing’s syndrome (*right panel*). Each set of three connected dots represents an individual. *Dashed line* is set at 80 pg/ml, i.e., upper limit of the normal range for immunometric chemiluminescent assay (Elecsys, Roche Diagnostics, Mannheim, Germany)

Measurement of ACTH has to take into account several factors, in particular its highly pulsatile secretory pattern, short plasma half-life—approx. 15 min [67]—and assay-related concerns. Foremost issue is the marked pulsatility of ACTH secretion, both in terms of pulse frequency and pulse amplitude [68], thus reliable estimates of plasma ACTH concentrations can be obtained only through multiple sampling (Fig. 3). Further, technical issues are known to affect plasma ACTH measurements, both in the preanalytical and analytical phase [69]. Appropriate use of anticoagulants, e.g., EDTA, siliconized glass tubes, rapid chilling, have been shown to improve ACTH analysis, although ACTH remains one of the less stable hormonal analytes [70]. As regards the analytical phase, the absence of an international ACTH reference standard means that each assay uses its own reference preparation and this leaves the issue of assay accuracy, i.e., true ACTH (1–39) concentrations, unresolved [71]. Indeed, considerable variability among ACTH measurements performed in different laboratories with different assay kits was recently reported [72], in line with results of external quality assessment programs showing 7–22 % coefficient of variation among ACTH assays [73]. To the clinician, the widely differing normal ranges reported by assay manufacturers, ranging from <46 pg/ml (<10 pmol/l) to 10–90 pg/ml (2.2–19.8 pmol/l), are an intuitive index of lack of assay standardization.

Demographic factors, i.e., age and sex, play a minor role although women present slightly lower ACTH concentrations than men, both in healthy individuals [74] and in patients with Cushing’s disease [3]. Further, oral contraceptives are associated with somewhat lower plasma ACTH levels [75].

**Fig. 4:**
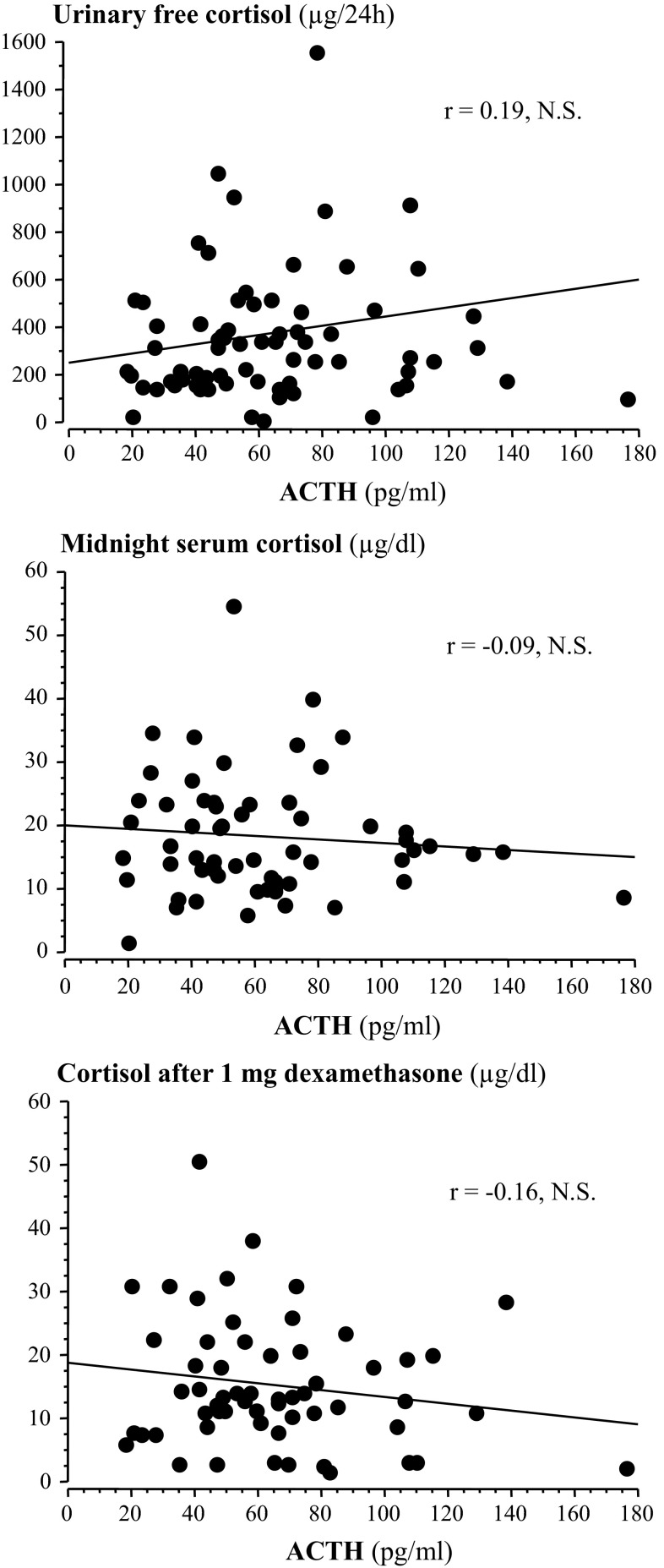
Lack of correlation between plasma ACTH and markers of hypercortisolism in patients with Cushing’s disease. Regression plot between ACTH and urinary free cortisol (*upper panel*), between ACTH and serum cortisol at midnight (*middle panel*) and between ACTH and cortisol after 1 mg dexamethasone

In Cushing’s disease, the ACTH-secreting tumor produces ACTH autonomously but, at variance with other pituitary tumors, e.g., GH- or prolactin-secreting, increased pituitary hormone levels are not mandatory to establish the diagnosis. Indeed, ACTH concentrations may well be comprised within the normal range in Cushing’s disease [25, 76] and, to a lesser extent, this is also true for ectopic ACTH secretion [25, 77]. In addition, plasma ACTH after surgery, again in contrast to other pituitary tumors, decreases but is not a clear indicator of remission [78] and, likewise, changes in plasma ACTH during medical therapy are less obvious responses to treatment [17, 18]. This suggests that measurement of ACTH per se is a poor indicator of corticotroph tumor activity, and, indeed, ACTH levels are poorly correlated with other markers of hypercortisolism, e.g., UFC, cortisol after low-dose dexamethasone (Fig. 4). It is worth recalling that in addition to marked amplification and greater disorderliness, ACTH secretion appears less synchronized with cortisol release in patients with Cushing’s disease [79].

In the differential diagnosis between ACTH-dependent and ACTH-independent Cushing’s syndrome, greater reliance is placed in accuracy of low rather than high ACTH measurements. Recent studies have shown, however, that reliability of low ACTH measurements is far from absolute and a consistent number of patients with adrenal adenoma, carcinoma, nodular dysplasia, or nodular hyperplasia present unsuppressed or even normal plasma ACTH levels [25, 80]. In fact, ACTH measurements proved misleading in up to 40 % of patients with adrenal Cushing’s syndrome [72, 76].

As regards assay methodology, comparison between competitive, single-antibody RIA and sandwich, two-site immunometric revealed that human plasma contains different ACTH species which can variably affect assay results. In fact, immunometric assays often yielded higher ACTH values than RIA in patients with Cushing’s disease [81, 82] whereas the opposite was observed in patients with ectopic secretion [81], in whom POMC and ACTH processing may follow alternative pathways. In most laboratories, RIA has been superseded by non-competitive radiometric or chemiluminescent assays but, even among the latter, some will yield up to 20 % higher measurements than others [83]. In fact, although correlation between measurements was statistically sound, deviances in the lower assay range [72, 82] may prove clinically significant for the differential diagnosis of Cushing’s syndrome.

As regards CRH testing and IPSS, both these procedures require comparison of ACTH measurements, i.e., baseline versus stimulated or central versus peripheral, thus a possible assay-related bias is unlikely to affect diagnostic accuracy [82]. Previous studies on CRH testing showed that repeat 100 µg human CRH administration to normal subjects yielded superimposable ACTH peak responses [84]; it has been our experience that ACTH responses in patients with Cushing’s disease, both in the active phase and after long-term remission are by and large similar in a given individual (Pecori Giraldi, unpublished data). In those rare cases in whom repeat IPSS was performed due to contrasting imaging and hormonal findings, test results proved comparable [85].

## Conclusions

Measurements of cortisol and ACTH in serum, urine, or saliva are subject to considerable variability, inherent to hormonal secretion and assay methodology. On the one side, irregularity in hormone secretion is accentuated in Cushing’s syndrome and complicates disease assessment. On the other side, assay platforms for urinary as well as salivary cortisol lack harmonization and measurement of ACTH plasma is susceptible to a variety of confounders. Measured concentrations of either hormone appear as an approximate marker of tumoral hypersecretion and disease activity and major efforts should be expended by the endocrinological community in order to close this gap and ameliorate soundness of cortisol and ACTH assays.

Altogether, interpretation of ACTH and cortisol measurements requires clinical expertise coupled with the knowledge that no single measurement is 100 % accurate for the diagnosis and, by inference, management of Cushing’s syndrome.
